# The Relationship between Nutrition in Infancy and Cognitive Performance during Adolescence

**DOI:** 10.3389/fnut.2015.00002

**Published:** 2015-02-11

**Authors:** Anett Nyaradi, Wendy H. Oddy, Siobhan Hickling, Jianghong Li, Jonathan K. Foster

**Affiliations:** ^1^School of Population Health, The University of Western Australia, Perth, WA, Australia; ^2^Telethon Kids Institute, The University of Western Australia, Perth, WA, Australia; ^3^WZB Berlin Social Research Center, Berlin, Germany; ^4^Faculty of Health Sciences, Centre for Population Health Research, Curtin University, Perth, WA, Australia; ^5^School of Psychology and Speech Pathology, Curtin University, Perth, WA, Australia; ^6^Neurosciences Unit, Health Department of Western Australia, Perth, WA, Australia; ^7^School of Paediatrics and Child Health, The University of Western Australia, Perth, WA, Australia

**Keywords:** nutrition, breastfeeding, cognitive performance, CogState, early childhood, adolescence, Raine study

## Abstract

**Objectives:** In this study, we aimed to investigate the long-term associations between breastfeeding duration during infancy, diet quality as measured by a diet score at 1 year of age, and cognitive performance during adolescence.

**Methods:** Participants (*n* = 717) were recruited from the West Australian Pregnancy Cohort (Raine) Study, a prospective longitudinal study of 2868 children and their families based in Perth, WA, Australia. Breastfeeding duration and an early diet score at age 1 year were used as the main predictor variables, while a computerized cognitive battery (CogState) was used to assess adolescents’ cognitive performance at 17 years. The diet score, which has seven food group components, was based on a 24-h recall questionnaire completed by the mother at 1 year of age. A higher diet score represents a better, more nutritious eating pattern. Associations between breastfeeding duration, diet score, and cognitive performance were assessed in multivariable regression models.

**Results:** Higher diet scores at 1 year representing better diet quality were significantly associated with faster reaction times in cognitive performance at 17 years [Detection Task (DET): β = −0.004, 95% CI: −0.008; 0.000, *p* = 0.036; Identification Task (IDN): β = −0.004, 95% CI: −0.008; 0.000, *p* = 0.027]. Breastfeeding duration (≥4 months) was also significantly associated with a shorter reaction time, but only for males (DET: β = −0.026, 95% CI: −0.046; −0.006, *p* = 0.010).

**Conclusion:** Nutrition in early childhood may have a long-term association with fundamental cognitive processing speed, which is likely to be related to enhanced brain development in the first year of life.

## Introduction

It is well known that the first 2 years of life represent a sensitive period for neurodevelopmental processes (1, 2). These processes are genetically programed but are also influenced by environmental factors, including nutrition (1, 3, 4). Optimal nutrition during this critical developmental period may have long-lasting positive effects on individuals’ later cognitive abilities (5).

Breastfeeding is a nutritional factor that has been widely researched and reviewed in relation to cognitive performance in children. Early reviews have not provided compelling evidence linking breastfeeding to cognitive development (6–8). However, a recent review (9) concluded that after adjusting for confounders, which strongly influence breastfeeding including socioeconomic status and maternal education or IQ, there was a consistent positive association between breastfeeding duration and IQ with a difference between breastfed and non-breastfed children of approximately 2–5 points in IQ. Most of the research conducted to date on these associations has examined the effect of breastfeeding on cognitive development during childhood (6–9), while less is known about whether positive longer-term associations between breastfeeding and cognitive capacity are present (10, 11). One study examined longer-term associations between breastfeeding and cognitive performance in children from 8 to 18 years of age in New Zealand. They found that a longer duration of breastfeeding (categorized as: not breastfed; breastfed for <4 months, 4–7 months, and ≥8 months) was positively associated with cognitive and academic performance, as measured by the Wechsler Intelligence Scale for Children, teachers’ rating of school performance, reading and mathematics tests, and high school outcomes (10). Another study, conducted in Northern Ireland, found that 11- to 16-year-old adolescents breastfed for more than 12 weeks, compared to those who were breastfed between 1 and 12 weeks, showed higher verbal, numerical, and reasoning IQ and performed better on the Raven’s Standard Progressive Matrices test (11). The first aim of our study was to evaluate evidence concerning the prospective association between breastfeeding duration and cognitive performance at 17 years.

It is important to determine whether a longer-term association exists between diet in the early years and cognitive capacity in adolescence. This was a secondary aim of our study. While the possible connection between cognitive development and single nutrients has been widely researched in children (5), fewer studies have undertaken a more comprehensive assessment of nutrition in early childhood and related this to cognitive capacity (12–15). Moreover, only one study that we are aware of has explored these links in adolescence (16). In the Avon Longitudinal Study of Parents and Children (ALSPAC), researchers identified four dietary pattern trajectories with respect to food intake between 6 and 24 months of age: “Healthy,” “Discretionary,” “Traditional,” and “Ready-to-Eat.” They found negative associations between the “Discretionary” diet (that included foods such as crisps and chocolate) and the “Traditional” diet (characterized by intake of meat, cooked vegetables, and puddings) and cognitive performance as measured by the Wechsler Abbreviated Scale of Intelligence (WASI) at the age of 15 years (16).

Previously in the Western Australian Pregnancy Cohort (Raine) Study, breastfeeding was positively associated with language development at 6 and 10 years of age (17, 18) and those children who had a better quality diet at 1 year manifested better language and reasoning ability at 10 years (19). In the current study, we hypothesized that longer breastfeeding duration and a better dietary score during infancy would be associated with better cognitive performance in adolescence.

## Materials and Methods

### Study population

Participants were recruited from The Western Australian Pregnancy Cohort (Raine) Study. Details of the study are described elsewhere (20). In brief, 2,900 women were initially recruited from King Edward Memorial Hospital (the major tertiary maternity hospital in Perth, WA, Australia) and surrounding private practices between 1989 and 1991. Women between 16 and 20 weeks’ gestation were randomly enrolled into the study to assess the effects of repetitive pregnancy ultrasounds. The initial cohort included a total of 2,868 babies [89.5% full-term (≥37 weeks gestation) and 10.5% pre-term infants (<37 weeks gestation)]. Families were followed up at regular intervals at ages 1, 2, 3, 5, 10, 14, and 17 years. Ethics approvals were obtained from The Human Ethics Committee at King Edward Memorial Hospital and/or Princess Margaret Hospital for Children (Perth, WA, Australia) for every Raine follow-up. In the current study, we analyzed breastfeeding and dietary data from the first 3 years of life as well as cognitive performance scores from the 17-year follow-up (undertaken between 2006 and 2009), when 717 adolescents (89% full-term gestational age) completed a computerized cognitive test battery.

### Predictor variables

#### Breastfeeding

Breastfeeding data were collected at 1–3 years of age, with data collection being retrospective in the previous year. The method used for collecting these data represents a valid and reliable estimate of breastfeeding initiation and duration (21). The age in months at which breastfeeding was discontinued was recorded, giving a continuous measure of breastfeeding duration. These data were also dichotomized as: <4 months or ≥4 months breastfeeding. We chose to include this binary breastfeeding variable in the analysis because at the time of data collection (1990–1995), the recommendation from the Word Health Organization (WHO) was that all babies should be exclusively breastfed from 4 to 6 months. This differs from the current recommendation that all infants should be exclusively breastfed for 6 months (22). (We did not take into account the introduction of solid foods.)

#### Diet score

At 1 year of age (i.e., between 1991 and 1993), primary caregivers completed a modified 24-h dietary recall questionnaire during the clinic visit (with assistance provided from a research nurse). Data were collected only once from each caregiver regardless of season. The collected dietary data were entered into the dietary analysis program FoodWorks^®^ (Professional Version 5, 2007, Xyris Software, Brisbane, QLD, Australia). As serving sizes were not consistently recorded, the nutritionists entering the data used each “eating occasion” to represent “one serving of food,” as reported in other publications (23, 24). For our study, a team of three nutritionists categorized 2,262 individual foods and drinks consumed by participants into 100 food groups. These were further categorized into 20 categories according to the Australian Guide to Healthy Eating (25).

The diet score derived from these data include seven dietary components from 20 broad food categories [i. whole grains, ii. vegetables, iii. fruits, iv. meat ratio (numerator: white meat, egg, other protein sources, denominator: red meat, processed meats), v. dairy, vi. snack foods, and vii. sweetened beverages] (Table S1 in Supplementary Material). Scoring was guided by the Youth Healthy Eating Index (YHEI) (26) and adapted to our study data (19, 27). Each of the seven components received a score between 0 and 10 according to the number of eating occasions of each food group. A higher score represented more eating occasions of foods from the categories of wholegrain, vegetables, fruits, protein/meat ratio, and dairy and less frequent consumption from the categories of snack foods and sweetened beverages. The number of age-appropriate servings/eating occasions was based on the Dietary Guidelines for Children and Adolescents in Australia (28), which are similar to the servings used in the YHEI. For example, an infant who consumed a “healthy” food component (such as fruit, vegetables, dairy, wholegrains, or a high meat ratio) three or more times a day (i.e., optimal number of eating occasions) received a score of 10 for that dietary component. If none of these “healthy” foods were consumed, a score of zero was achieved for that component. Conversely, an infant who did not consume snack foods or soft drinks received the highest score for that dietary component, while three or more eating occasions for either of these components received a zero score. These scores were then summed to determine an overall dietary score ranging from 0 to 70, with a higher score representing a more healthy eating pattern (19) (Table S1 in Supplementary Material). This overall score was subsequently rescaled for easier interpretation from 0 to 10, with a higher score representing a healthier eating pattern. In summary, a better diet score indicates higher consumption of fruit, vegetables, wholegrain, dairy, white meat, and legumes and less consumption of red and processed meats, soft drinks, and snack food. The diet score was found to be normally distributed and correlated well with the socio-demographic characteristics of the cohort (27). Furthermore, in a previous study, a better early diet score was associated with an improved cognitive performance in middle childhood (19).

### Outcome variables

The cognitive assessment was undertaken between 2007 and 2009, when participants were a mean age of 17.11 years (range: 16.01–18.32 years; SD: 0.22 years). Cognitive performance was measured by a computerized cognitive battery developed by CogState (CogState Ltd., Melbourne, Australia). CogState is a valid, reliable, and sensitive test designed to assess neurocognitive functioning across the lifespan (29, 30). CogState is suitable for different cultures and requires only minimal language skills (31). It has limited practice effects (32) and correlates well with conventional neuropsychological measures (Pearson’s *R* in the range.49–0.83) (33). In our study, four tasks were chosen from the CogState battery (summarized in Table S2 in Supplementary Material), from which four main outcome variables were analyzed. The Detection Task (DET) was the least complex task in the test battery; a playing card was presented on the screen and participants were required to indicate by pressing a key as quickly as they could as soon as the card turned over. This task measures basic psychomotor speed. The principal outcome variable was reaction time. The Identification Task (IDN) is a simple choice response task; in this task, the participants were required to indicate whether the card that turned over is red (using “yes” or “no” keys on the computer keyboard). This task assesses psychomotor speed and visual attention. The principal outcome measure was reaction time. In the One Card Learning Task (OCL), participants were required to indicate by pressing a key if they had seen the card that appeared on the screen before within the same task. The task measures visual learning and memory. The principal outcome variable was the proportion of correct responses. In the Continuous Paired Association Learning Task (CPAL), participants were asked to recall the correct spatial location of a previously presented object. This task evaluates visual and spatial learning and memory. The principal outcome measure was the total number of errors made.

### Confounding factors and covariates

Potential confounding factors included in the regression models were as follows: maternal age, maternal education, family income, and the biological father’s presence in the family. Gender was also included in the regression models.

Maternal data were collected during pregnancy. Maternal age was classified as a continuous variable. Maternal education was categorized as follows: none, trade certificate/apprenticeship, non-degree professional registration, college/TAFE diploma, university degree, and other.

Family data were collected at the 1-year follow-up between 1990 and 1993. Family income was coded into five categories: <AUD $7,000; $7,000–$11,999; $12,000–$23,999; $24,000–$35,000; and ≥$36,000. The presence of the biological father within the family was categorized as: “yes” and “no.”

### Statistical analysis

Data were scrutinized for missing values, invalid data and normality. We excluded participants who failed to complete 75% of the trials and did not pass the integrity checks for the DET, IDN, and OCL tasks. This procedure was followed as per CogState guidelines (29). Integrity check criteria were as follows: DET speed scores <IDN speed scores and DET accuracy >90%; IDN accuracy >80%; OCL accuracy >50%. The distributions of the total error rate for the CPAL task were positively skewed; therefore, a log10 transformation was applied to the data to achieve normality. The reaction time scores for the DET and IDN were also log10 transformed and the square root of the proportion of correct responses for the OCL task was arcsine transformed. These standard procedures with respect to the cognitive date were undertaken by CogState Ltd., Melbourne, VIC, Australia.

We analyzed the data in three steps. Group comparisons, using Pearson chi-square tests for categorical variables and independent sample *t*-tests for continuous variables, were initially undertaken to compare characteristics of participants who did or did not complete the CogState cognitive battery. Means of outcome variables were compared between breastfeeding categories, using one-way ANOVAs, and correlations were computed between diet scores and the outcome variables. Lastly, General Linear Modeling was implemented to perform multivariable regression analyses to examine the associations between breastfeeding duration (as a continuous variable and categorized as <4 months, ≥4 months) and nutrition in early childhood (measured by the continuous diet score at age 1 year) with the CogState outcomes as described previously. These analyses were performed on each cognitive outcome separately, adjusting for relevant confounders and covariates including gender, maternal age, maternal education, family income, and presence of the biological father in the family. To assess a potential moderating effect of gender, initially gender × diet and gender × breastfeeding interaction terms were included in all models, followed by a separate analysis undertaken for males and females if a significant interaction was present. All analyses were performed using IBM SPSS Statistics 19 with α = 0.05 set as the criterion for statistical significance.

## Results

### Sample attrition

Table 1 shows the relevant descriptive statistics concerning the participants who took part in CogState testing. Family income was >AUS$36,000 in 37% of participants at age 1, while 40% of the mothers did not have any formal qualifications and only 13% reported possession of a university degree. Breastfeeding continued for more than 4 months in 69% of the participants. Adolescents who participated in the CogState cognitive battery at the 17-year follow-up were born to mothers who were older (*p* < 0.001), more educated (*p* < 0.001), had a greater probability of living with the child’s biological father (*p* < 0.008), had higher family incomes (*p* < 0.001), and were more likely to have breastfed their child for a longer period (*p* < 0.001), than study participants who did not take part (Table S3 in Supplementary Material).

**Table 1:** **Descriptive statistical data for participants in CogState at 17 years of age (*n* = 717) in the Western Australian Pregnancy Cohort (Raine) Study**.

	CogState participants *n* = 717	
Continuous variables	Mean (range)	SD
Detection Task (DET) *n* = 496	275.42 (204.17–602.56)	1.15
Identification Task (IDN) *n* = 664	436.52 (323.59–870.96)	1.15
One Card Task (OCL) *n* = 669	0.85 (0.71–0.99)	0.10
Continuous Paired Association Learning Task Part 1 and Part 2 (CPAL) *n* = 717	30.94 (0–235)	29.86
Diet score age 1	6.58 (3–10)	1.45
Maternal age (pregnancy)	28.72 (15–43)	5.79
Breastfeeding (continuous variable)	6.74 (0–12)	4.34

**Categorical variables**	***N***	**%**

Breastfeeding
<4 months	210	30.6
≥4 months	476	69.4
Gender of the child		
Female	343	47.8
Male	374	52.2
Maternal education (pregnancy)
None	281	39.9
Trade certificate/apprenticeship	54	7.7
Non-degree professional registration	85	12.1
College/TAFE diploma	147	20.9
University degree	93	13.2
Other	45	6.4
Family income (age 1)
<$7,000	11	1.7
$7,000–$11,999	48	7.3
$12,000–$23,999	152	23
$24,000–$35,999	205	31
≥$36,000	246	37.2
Father living with family (age 1)
Yes	608	88.9
No	76	11.1

### Breastfeeding

Unadjusted mean scores for each cognitive measure according to breastfeeding categories are shown in Table 2. There were significant differences in mean reaction times on the DET (<4 months: 280.67 ms; ≥4 months: 270.33 ms; *p* = 0.007) and the IDN (<4 months: 448.44 ms; ≥4 months: 434.61 ms; *p* = 0.010) tasks. In the multivariable regression models, after adjusting for confounders and covariates (Table 3), there was a significant interaction between gender and breastfeeding on the DET task, i.e., whether or not a child was breastfed for 4 months or longer had a differential effect on the reaction time on DET task depending on the child’s gender (*F* = 4.40, *p* = 0.037). There was also a significant interaction between gender and breastfeeding on DET task performance when breastfeeding was treated as a continuous variable (*F* = 4.53, *p* = 0.034). Therefore, separate analyses for males and females were conducted on the DET task. These gender-specific analyses revealed that for males there was a significant association between breastfeeding for 4 months or longer and faster reaction time on the DET task (β = −0.023, 95% CI: −0.043; −0.002, *p* = 0.029), but not for females (β = 0.004, 95% CI: −0.013; 0.021, *p* = 0.642). The associations between diet and breastfeeding as a continuous variable were not significant in either males (β = −0.002, 95% CI: −0.004; 0.000, *p* = 0.079) or females (β = 0.001, 95% CI: −0.001; 0.003, *p* = 0.441).

**Table 2:** **Unadjusted mean scores (and SD) for each cognitive measure according to breastfeeding category, and Pearson’s correlation between diet score at age one and each cognitive measure**.

CogState tasks	Breastfeeding				*p*
	<4 months		≥4 months	
	*N*	M (SD)	*N*	M (SD)	
DET (ms)^a^	129	280.67 (1.15)	344	270.33 (1.14)	0.007
IDN (ms)^a^	190	448.44 (1.16)	445	434.61 (1.14)	0.010
OCL (proportion correct)^b^	192	0.85 (0.10)	450	0.85 (0.10)	0.218
CPAL (count)^a^	210	31.54 (30.15)	476	29.92 (28.66)	0.067

	**Diet score age 1^c^**				
	***N***	***R***			

DET (ms)^a^	440	−0.12			0.014
IDN (ms)^a^	598	−0.11			0.008
OCL (proportion correct)^b^	601	0.05			0.180
CPAL (count)^a^	642	−0.06			0.113

Means and SD are calculated on untransformed data. (Those variables that had been transformed by CogState were back transformed.)

*DET, Detection Task; IDN, Identification Task; OCL, One Card Learning Task; CPAL, Continuous Paired Association Learning Task*.

*^a^Lower score = better performance*.

*^b^Higher score = better performance*.

*^c^Higher score = better diet*.

**Table 3:** **Multiple Regression analyses evaluating (a) breastfeeding and cognitive performance at 17 years of age and (b) diet score at 1 year and cognitive performance at 17 years of age in the Western Australian Pregnancy Cohort Study adjusted for sex, family income (age one), father’s presence in the family (age one), maternal age (pregnancy), and maternal education (pregnancy)**.

Diet	DET^a^		IDN^a^		OCL^b^		CPAL^a^	
	β (95% CI)	*p*	β (95% CI)	*p*	β (95% CI)	*p*	β (95% CI)	*p*
**Breastfeeding**								
<4 months (reference)
<4 months	−0.010 (−0.023; 0.002)	0.133	−0.010 (−0.023; 0.003)	0.080	0.011 (−0.009; 0.030)	0.283	−0.040 (−0.115; 0.035)	0.295
Male
<4 months (reference)								
≥4 months	−0.023 (−0.043; −0.002)	0.029						
Female
<4 months (reference)							
≥4 months	0.004 (−0.013; 0.021)	0.642					
**Breastfeeding (continuous; 0–12)**	−0.001 (−0.002; 0.001)	0.352	−0.001 (−0.002; 0.001)	0.200	0.002 (−0.001; 0.004)	0.152	−0.002 (−0.010; 0.006)	0.628
Male	−0.002 (−0.004; 0.000)	0.079						
Female	0.001 (−0.001; 0.003)	0.441						
**Diet score age 1 (0–10)^c^**	−0.004 (−0.008; 0.000)	0.036	−0.004 (−0.008, 0.000)	0.027	0.005 (−0.002; 0.011)	0.142	−0.007 (−0.031; 0.017)	0.580

*DET, Detection Task; IDN, Identification Task; OCL, One Card Learning Task; CPAL, Continuous Paired Association Learning Task*.

*^a^Lower score = better performance*.

*^b^Higher score = better performance*.

*^c^Higher score = better diet*.

### Diet scores at age 1

Pearson’s correlations between diet score at 1 year and each cognitive measure are presented in Table 2. Negative correlations between the diet score and the response times on the DET (*r* = −0.117, *p* = 0.014) and IDN (*r* = −0.108, *p* = 0.008) tasks were observed. After adjustment, a higher diet score at 1 year was associated with faster reaction times on the DET (β = −0.004, 95% CI: −0.008; 0.000, *p* = 0.036) and IDN (β = −0.004, 95% CI: −0.008; 0.000, *p* = 0.027; Table 3) tasks. Gender × diet interactions were not significant in any models examining diet score.

## Discussion

In this study, we examined the long-term impact of breastfeeding and nutrition in early childhood on the cognitive ability of adolescents. We found that boys who had been breastfed for 4 months or longer showed better cognitive performance with regard to psychomotor speed reflected by faster reaction times on the CogState DET. This association remained significant after adjustment for the socioeconomic covariates maternal age and education, family income, and the presence of the biological father in the family. We did not find any association between breastfeeding and cognitive performance in girls. We found that better diet quality at 1 year was associated with faster reaction time on the CogState DET and IDN task in both boys and girls.

The effects of breastfeeding on cognitive capacity in adolescence (11, 34–36) and adulthood have been studied previously (37, 38). Our results are similar to the results reported by Kafouri et al. (34). These researchers showed a positive association between a longer duration of breastfeeding and full scale and performance (but not verbal) IQ, as measured on the Wechsler Intelligence Scale for Children III (WISC-III). The CogState test battery does not measure IQ *per se* and we were not able to compute an overall composite score of test performance (as the measurement parameters were different across the CogState tasks). However, we found that one of the key CogState outcome variables measuring psychomotor speed (an important element of the “performance” component of intelligence) was significantly associated with breastfeeding duration in boys. In the study conducted by Kafouri et al. (34), a longer breastfeeding duration was associated with higher performance IQ. This component of IQ is measured by tasks that evaluate how accurately and quickly people can perform; this capacity is conceptually linked to psychomotor speed. Therefore, there appears to be consistency between our current findings and those reported by Kafouri et al. (34).

Gender benefits related to breastfeeding have been reported by Isaacs et al. (35). In their study, an association was observed between IQ, brain volume (specifically white matter volume), and breast milk intake in premature infants who were subsequently evaluated during adolescence. This association was highly significant in males, but not in females. Isaacs et al. (35) reported an association predominantly observed in males, on verbal and full scale IQ (measured by the WISC) but not on performance IQ. Gender differences have previously been noted by Oddy et al. (39) in the Raine cohort; i.e., longer breastfeeding duration was significantly associated with better academic performance (mathematics and spelling) at age 10 years in boys only. Our study has shown that this gender difference continues to 17 years with regard to cognitive test performance. It should be noted that, in contrast to the findings of Isaacs et al. (35), the gender difference observed in our study was reflected in a measure conceptually related to “performance” IQ rather than to “verbal” IQ.

Gender differences in neural development, brain structure, and neurocognitive capacity have been reported in numerous studies, and confirmed by neuroimaging (40–42). The difference in males and females’ neurocognitive development may be attributed to sex hormones, as receptors for these hormones can be found across several brain regions (43). Other factors potentially mediating the gender differences observed in our study could include the neuroprotective effect of the sex hormone estradiol in females (44). The lack of estradiol in males is consistent with the notion of greater vulnerability of the male brain to external events and the nutritional environment; therefore, it is plausible to suggest that a shorter duration of breastfeeding would exert a more detrimental effect on boys’ neurocognitive development than on girls’ cognitive capacity.

To our knowledge, there is only one published study that has examined the long-term effect of dietary patterns during the early years on cognitive functioning. Smithers et al. (16) analyzed dietary pattern trajectories from 6 to 24 months of age and cognitive performance at age 8 and 15 years. These researchers observed weak but positive associations between early diet and cognitive functioning measured later in life. These findings are supported by our study, whereby a better diet quality during the infant/toddler years was associated with higher levels of cognitive performance in adolescence.

We showed that performance on the DET was significantly associated with breastfeeding duration in boys and performance on the DET and IDN task was significantly associated with diet at 1 year in the whole cohort. A possible explanation of these results concerns the ontogeny of different neurocognitive functions. Specifically, the DET and IDN tasks are among the most “process pure” tasks in cognitive terms from those tests in the CogState test battery that were applied in the current study. These tasks evaluate elementary psychomotor functioning and attention using reaction time as the key performance parameter. The psychomotor functions underlying reaction time represent some of the earliest capacities to develop, i.e., in terms of the fundamental cognitive building blocks that are subserved by the brain (45). More complex cognitive capacities emerge and develop until late adolescence and early adulthood (46), aligned with the maturation of specific brain areas (for example, the frontal lobes) over a similar time frame (4).

With respect to biological mechanisms underlying the current findings, the long-chain polyunsaturated fatty acids (LCPUFAs) – specifically omega-3 fatty acid docosahexaenoic acid (DHA) in breast milk – are considered to be key factor/s that may increase the intelligence of breastfed individuals. However, recent Cochrane reviews did not support this hypothesis, as significant associations between LCPUFA supplementation and cognitive development in term infants (47) or pre-term infants (48) were not found. In contrast, another review by Hoffman et al. (49) suggested that supplementation with a similar level of DHA to breast milk in formula milk resulted in higher cognitive performance in young children who were born full-term. Further, Issacs et al. (35) proposed that cholesterol (which is a significant component of breast milk) may exert a beneficial influence on children’s cognitive development through breastfeeding. Cholesterol is important in neuronal myelination and has been implicated in adult cognitive performance (35).

We found that a healthier diet during infancy may exert long-lasting cognitive benefits. The mechanism/s underlying these effects are likely to be linked to the high nutritional sensitivity of brain development in the critical early period of life. Specifically, the brain reaches 80% of its adult weight during the first 2 years of life due to relatively fast growth (1, 2). Furthermore, during the first year of life, structural and metabolic changes (including increased glucose metabolism, enhanced capillary density, and dendritic field and white matter myelination) commence in the cortex, reflective of early maturational processes (50–52).

Some of the strengths of our study include the use of a prospective design in a large, well-researched cohort with the ability to study the long-term effects of breastfeeding and nutrition in early childhood on cognitive capacities in adolescence. Cognitive functioning was evaluated using a computerized cognitive battery (CogState) representing a valid, reliable, and sensitive method for detecting differences in cognitive performance (29, 30).

Our study also has some limitations. First, the semi-quantitative dietary data that we obtained may not accurately represent the child’s usual diet. Second, although we did not have access to data on maternal intelligence *per se*, we adjusted for maternal education, which has been shown to be a reliable surrogate of mother’s intelligence (53). Third, due to the lack of CogState data for the entire cohort, we were only able to analyze data from the subset who completed CogState testing. These individuals differed significantly in socio-demographic characteristics from non-participants as specified earlier. A more representative sample of the cohort would contain greater variation in predictor and outcome variables. Therefore, the impact of breastfeeding and early diet on cognitive function in adolescence may be biased conservatively. We adjusted for the most relevant socio-demographic factors, maternal age, maternal education, family income, and the biological father’s presence in the family, but we also acknowledge other confounding not adjusted for. Finally, because this is an observational study, we cannot infer causality between breastfeeding/diet and cognitive outcomes.

In summary, we observed that nutrition in early life (including breastfeeding and diet at 1 year) manifested a significant association with cognitive performance in adolescents, specifically on more fundamental cognitive tasks. We identified a gender difference, indicating that breastfeeding for 4 months or longer was particularly important for boys in relation to long-term cognitive development. Further research is needed in this important area; for example, using structural and functional neuroimaging to evaluate the putative neurological mechanisms linking nutrition to neurocognitive development, and the specific biological mechanisms underlying the gender differences noted in our study.

## Conflict of Interest Statement

The authors declare that the research was conducted in the absence of any commercial or financial relationships that could be construed as a potential conflict of interest.

## Supplementary Material

The Supplementary Material for this article can be found online at http://www.frontiersin.org/Journal/10.3389/fnut.2015.00002/abstract

## Abbreviations

CPAL, continuous paired association learning task; DET, detection task; IDN, identification task; OCL, one card learning task.
