# Supplementary material for: The Relationship between Nutrition in Infancy and Cognitive Performance during Adolescence
Source: Front Nutr. 2015 Feb 11;2:2. doi: 10.3389/fnut.2015.00002 (PMC4451795; doi:10.3389/fnut.2015.00002)
Supplement: Supplementary file 1 [file Table_1.PDF]

Appendix 1: Number of eating occasions and their scoring in the diet score [source: (19)]

Components of the diet score	Example foods included in each component	Eating occasions 0	1	2	≥ 3	Score range of individual components
1. Wholegrain	High fibre breakfast cereals, crackers, pasta and rice. Wholemeal and wholegrain breads and grains.	0	5	10	10	0 – 10
2. Vegetables	Broccoli, cauliflower, cabbage, celery, carrot.	0	3.3	6.7	10	0 – 10
3. Fruit	Apple, orange, banana, raspberries, pineapple, grapes.	0	3.3	6.7	10	0 – 10
4. Meat Ratio	Numerator: Poultry, fish, eggs, legumes, nuts, seeds Denominator: red meat and processed meat	0	5	10	10	0 – 10
5. Dairy	Milk, cheese, yoghurt, custard, ice-cream, cream and soy alternatives.	0	3.3	6.7	10	0 – 10
6. Snack foods	Savoury/salty snacks: chips, crisps, popcorn, crackers, nachos. Sweet/sugary snacks: cakes, pastries, sweet biscuits, chocolates, lollies, jellies, icy poles	10	6.7	3.3	0	0 – 10
7. Sweetened beverages	Soda (soft drink), cordial and fruit drink.	10	6.7	3.3	0	0 – 10
Diet Score						0 – 70
