# Supplementary material for: The Relationship between Nutrition in Infancy and Cognitive Performance during Adolescence
Source: Front Nutr. 2015 Feb 11;2:2. doi: 10.3389/fnut.2015.00002 (PMC4451795; doi:10.3389/fnut.2015.00002)
Supplement: Supplementary file 2 [file Table_2.PDF]

Appendix 2: CogState tasks

CogState Tasks	Task descriptions	Main outcome measures and interpretation
Detection Task (DET)	Participants have to respond when the card is turned over	Mean of the $\log_{10}$ transformed reaction times in milliseconds for correct responses<br>Lower score = better performance
Identification Task (IDN)	Participants have to indicate whether the card that turned over is red	Mean of the $\log_{10}$ transformed reaction times in milliseconds for correct responses<br>Lower score = better performance
One Card Task (OCL)	Participants have to indicate if they have seen the card before in the task	Arcsine transformation of the square root of the proposition of correct responses<br>Higher score = better performance
Continuous Paired Association Learning Task Part 1 and Part 2 (CPAL)	Participants have to find the correct location of the object that match with the object in the centre	Total number of errors made on five trials in Part 1 and Part 2 (the two scores are averaged)<br>Lower score = better performance
