# Supplementary material for: The Relationship between Nutrition in Infancy and Cognitive Performance during Adolescence
Source: Front Nutr. 2015 Feb 11;2:2. doi: 10.3389/fnut.2015.00002 (PMC4451795; doi:10.3389/fnut.2015.00002)
Supplement: Supplementary file 3 [file Table_3.PDF]

Appendix 3: Differences in sociodemographic and dietary characteristics between CogState participants (with at least one valid CogState tasks score: n = 717) and non-participants (all other Raine study members)

	Participants in CogState tasks	Non-participants	
Continuous variables	***Mean (SD)***	***Mean (SD)***	***P value****
Diet score age one	6.58 (1.45)	6.48 (1.45)	0.173
Maternal age	28.72 (5.79)	27.19 (5.92)	<0.001
Categorical variables	***% (n)***	***% (n)***	***P value*****
Breastfeeding			
<4month	30.6 (210)	40.7 (705)	<0.001
≥4 month	69.4 (476)	59.3 (1028)	
Gender of the child			
Female	47.8 (343)	49.8 (1071)	0.365
Male	52.2 (374)	50.2 (1080)	
Maternal education (pregnancy)			
none	281 (39.9)	1168 (55.6)	<0.001
trade certificate/ apprenticeship	54 (7.7)	180 (8.6)	
non-degree professional registration	85 (12.1)	161 (7.7)	
collage/ TAFE diploma	147 (20.9)	301 (14.3)	
university degree	93 (13.2)	187 (8.9)	
other	45 (6.4)	102 (4.9)	
Family income (age one)			
< AU$7,000	1.7 (11)	1.1 (18)	<0.001
	7.3 (48)	9.5 (157)	
AU$7,000 - AU$11,999	23 (152)	33.8 (559)	
	31 (205)	27.9 (461)	
AU$12,000 - AU$23,999	37.2 (246)	27.7 (457)	

AU$24,000 - AU$35,999			
>=AU$36,000			
Father living with family (age one)			
Yes	88.9 (608)	84.7 (1460)	0.008
No	11.1 (76)	15.3 (264)	

*Independent-samples T test

**Pearson chi-square test
